# Interventions and outcomes of COVID-19 patients in a community hospital–A single center study comparing the first and second waves

**DOI:** 10.1371/journal.pone.0279208

**Published:** 2022-12-16

**Authors:** Pamela Lamisi Alebna, Jessica Chung, Muhammad Rashid, Davina Hoban, Mabel LaForgia, Surendra Khera, Michael Loftus

**Affiliations:** Department of Internal Medicine, Jersey City Medical Center, Jersey City, New Jersey, United States of America; City University of New York, UNITED STATES

## Abstract

**Background:**

We have had 3 coronavirus-related pandemics in the last two decades. Each has brought significant toll and with each case there was no cure. Even as vaccines have been developed for the current strain of the virus thereby increasing the prospects of bringing transmissions in communities to a minimum, lessons from this pandemic should be explored in preparation for future pandemics. Other studies have looked at differences in characteristics of patients and mortality rates between the first two waves. In our study we not only identify the differences in outcomes but also explore differences in hospital specific interventions that were implemented at Jersey City Medical Center, NJ, a community-based hospital.

**Aim:**

The aim of this study is to assess the differences between the first two waves of the COVID -19 pandemic in terms of management and outcomes to help identify any key lessons in the handling of future pandemics. We compared the population demographics, interventions and outcomes used during the first two waves of COVID-19 in a community-based hospital.

**Methods:**

This is a retrospective single-center cross-sectional study including Laboratory confirmed COVID-19 patients requiring oxygen supplementation admitted at Jersey City Medical Center during the first wave (April 1 to June 30, 2020) and the second wave between (October 1, 2020, and January 1, 2021). The Chi-squared test was used to assess the relationship between categorical variables and the T- test for continuous variables. A Logistic regression model was built comparing the second to the first wave while accounting for important covariates.

**Results:**

There was a combined total of 473 patients from both waves. Patients in the first wave were older (66.17 years vs 60.38 years, p <0.01), had more comorbidities (2.75 vs 2.29, p 0.003), had more severe disease (50% vs 38.78% p of 0.002), had a longer length of stay (14.18 days vs 8.77 days, p <0.001) and were more likely to be intubated (32.49% vs 21.9 4%, p 0.01). In the univariate model, the odds of mortality in the second wave compared to the first wave was 0.63 (CI, 0.41–0.96) and 1.73 (CI, 0.65–4.66) in the fully adjusted model.

**Conclusion:**

Overall, there was no statistically significant difference in mortality between the two waves. Interventions that were noted to be significantly different between the two waves were, increased likelihood of mechanical intubation in the first wave and increased use of steroids in the second wave compared to the first.

## Introduction

The 2019 coronavirus (COVID-19), also known as SARS-CoV-2 has infected over 565 million people worldwide with over 6.3 million dead since it was first detected [1]. COVID-19 is highly pathogenic and virulent, and it spreads very quickly through human-to-human contact [2–4].

It occurred in several peaks and waves [5,6]. Many countries have experienced at least three waves of the pandemic to date.

In the early days of the COVID-19 pandemic intensivists were inundated with record number of patients requiring ventilator support and thus ICU admissions [7,8]. One can assume that the experience gained during the first wave may have contributed to a better management and outcome among critically ill COVID-19 patients admitted during the second wave [9].

The trend in cases was characterized by an initial surge in the spring followed by a decline and a resurgence in the fall of 2020 [10]. Studies have shown that in general, the overall outcomes in the second wave were better than the first [11,12], with a few suggesting otherwise [13,14].

The findings of one study comparing the first and second waves in Japan indicated that in the first wave, the medical system was under greater strain with more severe cases on admission^,^ [15]. In the second wave, there was a smaller proportion of severe cases, patients were younger with fewer underlying diseases and mortality rate was lower [10].

The case fatality rate (CFR) is the reported number of COVID‐19 deaths divided by the total number of cases. It is an important indicator to quantify the severity of disease or treatment efficacy. One study reported the CFR of 53 countries or regions with the highest COVID‐19 death tolls. Of them, 43 had lower CFR estimates in the second wave than in the first wave [16].

Some other studies showed that despite a better understanding of COVID-19 with significant treatment modification including systematic and early administration of glucocorticoids as well as intermediate/full dose thromboprophylaxis, the researchers did not observe any decrease in ICU mortality, with still half of the patients dying in the ICU [17]. Some did however show a lower rate of thrombotic events observed during the second wave, which the researchers felt was likely inherent to the increased use of thromboprophylaxis [18].

In our study, we hypothesize that, patients with severe COVID-19 infection will have a shorter length of stay in the second wave compared to the first, mortality rates in the second wave will be better than the first, and hospital specific interventions improved outcomes in the second wave.

## Methods

### 2.1 Study sample

This is a retrospective single-center cross-sectional study.

#### Inclusion criteria

Patients were eligible for inclusion in this research study if they were male or female age >18, admitted to Jersey City Medical Center (JCMC), in Jersey City, NJ, diagnosed with COVID-19 via PCR testing and requiring supplemental oxygen. Patients admitted during the time periods of April 1- June 30, 2020, were considered the first wave and October 1, 2020—January 1, 2021, as the second wave.

#### Exclusion criteria

Patients were excluded if they were admitted for COVID-19 and not requiring oxygen supplementation.

### 2.2 Data collection

Data was collected from the Jersey City Medical Center COVID-19 patient registry. Data was fully anonymized before access. The Institutional Review Board (IRB) at Jersey City Medical Center approved the study and waived requirement for informed consent. Data collected included information on the following: period of hospitalization for COVID-19 pneumonia in either the first or second wave, hospital specific interventions (doses of steroid management), all-cause mortality, length of hospital stay, disease severity using the WHO ordinal scale for clinical improvement [19], discharge from the hospital (to home or rehabilitation facility), demographic factors (age, sex, ethnicity), health-related risk factors (BMI), steroid use (type, dose, duration), other therapies (Remdesivir, Convalescent plasma, Therapeutic Anticoagulation, Monoclonal antibodies), and the type and number of comorbid conditions.

We estimated a sample size of 370 determined by assuming a power of 90% at a two-sided alpha level of 0.05 to determine a minimum detectable OR of 0.12.

#### The first and second wave

We defined the first wave as the period from April 1 to June 30 2020, based on in-hospital admission data as well as trends in worldwide transmission [20]. The second wave was from October 1, 2020, to January 1, 2021.

#### Outcome

The primary outcome of interest was all-cause mortality. Even though data was collected on patient discharge disposition, either to home, long term acute care facility, subacute or acute rehabilitation facility the main outcome variable was categorized as either alive or expired to increase power in our analysis.

### 2.3 Statistical analysis

#### Descriptive statistics

Analysis began with data inspection and descriptive statistics of the individual covariates. We used the one-in-ten rule to estimate the maximum number of predictors to include in our model. Normality of continuous variables was assessed using the Shapiro-Wilk test, as well as test for skewness and kurtosis. Chi squared test was used for relationship between two categorical variables.

#### Model building

Key variables that could act as predictors were selected a priori based on subject matter knowledge, variables we deemed would directly affect outcome. A purposeful selection approach was used in determining potential covariates to include in a logistic regression model [21]. The main outcome was assessed initially with an unadjusted model and then with a multivariable model. The main outcome was assessed with 2 models: model 1 included the period of hospitalization only i.e., either first or second wave, model 2 included period of hospitalization with additional covariates of treatment with therapeutic anticoagulation (yes or no), number of comorbidities present, duration of stay in hospital (in days), treatment with steroids (yes or no), treatment with convalescent plasma (yes or no), mechanical ventilation (yes or no). Interactions with race were analyzed.

In all analyses a two tailed p value of <0.05 was considered statistically significant. All statistical analyses were performed with SAS 9.4.

## Results

### 3.1 Exploratory analysis

In total there were 473 patients, 277 in the first wave and 196 in the second wave. The demographic characteristics of participants are summarized in Table 1. The mean age of patients in the first wave was 66.17(SD 14.48) and 60.38(SD 16.28) in the second wave, the difference in age was statistically significant (p < .001). There was a significant difference observed in the use of steroid, 19.13% in the first wave and 92.86% in the second wave (p < .0001). 54.87% of patients in the first wave, were given therapeutic anticoagulation and 54.08% in the second wave (p 0.86). Patients were also more likely to be intubated in the first as compared to the second wave, 32.49% and 21.94% respectively (p 0.012). The race and mortality interaction term was not significant, hence there is no evidence of effect modification.

**Table 1:** Baseline characteristics of patients.

Characteristics	First wave N = 277	Second wave N = 196	p value
**Age**	66.18 ± 14.48	60.38 ± 16.28	< .001
**Sex (n, %)**			0.94
**Male**	162(58.48)	114(58.16)	
**Female**	115(41.52)	82(41.84)	
**Race (n, %)**			0.02
**Asian**	58(21.01)	26(13.27)	
**Black**	80(28.99)	44(22.45)	
**White**	51(18.48)	39(19.90)	
**Hispanic**	76(27.54)	74(37.76)	
**Other**	11(3.99)	13(6.63)	
** **BMI >30 (n, %)**			0.09
**<30**	14(5.05)	18(9.23)	
**≥30**	263(94.95)	177(90.77)	
**Disease Severity (n, %)**			0.020
**Mild**	1 (0.37)	0(0)	
**Moderate**	133 (49.63)	120 (61.22)	
**Severe**	134 (50.00)	76 (38.78)	
Number of Comorbidities*	2.75 ± 1.66	2.29 ± 1.65	0.003
**Steroids (n, %)**			< .001
**Yes**	53 (19.13)	182 (92.86)	
**No**	224 (80.87)	14 (7.14)	
**Convalescent plasma (n, %)**			0.151
**Yes**	52 (18.77)	27 (13.78)	
**No**	225 (81.23)	169 (86.22)	
**Therapeutic anticoagulation (n, %)**			0.86
**Yes**	152 (54.87)	106 (54.08)	
**No**	125 (45.13)	90 (45.92)	
**Remdesivir (n, %)**			< .001
**Yes**	15(5.42)	126(64.29)	
**No**	262(94.58)	70(35.71)	
**Mortality (n, %)**			0.03
**Died**	88(31.77)	45(22.96)	
**Alive**	189(68.23)	151(77.04)	
**LOS (mean ± SD days)**	14.18 ± 14.50	8.77 ± 8.45	< .001
**Invasive Mechanical Ventilation (n, %)**			0.012
**Yes**	90 (32.49%)	43 (21.94%)	
**No**	187 (67.51%)	153 (78.06%)	

*Comorbidities listed include- Diabetes, Hypertension, Hyperthyroidism, Dementia Asthma, CVA, CAD, COPD, Rheumatoid Arthritis, Chronic Kidney Disease.

** LOS indicates Length of Hospital Stay.

***Percentages may add up to >100% due to approximation ***.

### 3.2 Univariate and multivariable models

We present unadjusted ORs of disease severity, patient characteristics and mortality in Table 2. Not surprising, the odds of dying were lower 0.01 (CI, 0.001–0.97) in patients with moderate disease compared to patients with severe disease. There was no significant difference in odds ratio between patients who had received steroids verses those who had not and same for patients who had received Remdesivir. Therapeutic anticoagulation, convalescent plasma and the use of mechanical ventilation were all associated with lower odds of mortality in the unadjusted models, presented in Table 2. In the unadjusted model we found that the odds of dying were lower in the second wave compared to the first 0.63 (CI, 0.41–0.96), Table 3. This difference reversed after adjusting for a comprehensive set of potential confounders, including age, number of comorbidities present, length of stay, and therapeutic anticoagulation. The other covariates include steroid use, convalescent plasma and mechanical ventilation; however, this difference was not statistically significant.

**Table 2:** Unadjusted OR of disease severity/interventions and mortality.

Characteristics	OR	Confidence Interval	
***~*Disease Severity**			
**Moderate**	0.01	0.001	0.97
**Severe**	Ref	Ref	Ref
**Steroids**			
**No**	1.092	0.73	1.64
**Yes**	Ref	Ref	Ref
**Convalescent plasma**			
**No**	0.508	0.307	0.841
**Yes**	Ref	Ref	
**Therapeutic anticoagulation**			
**No**	0.18	0.11	0.30
**Yes**	Ref	Ref	Ref
**Remdesivir**			
**No**	0.99	0.64	1.54
**Yes**	Ref	Ref	Ref
**Mechanical Ventilator Use**			
**No**	0.028	0.016	0.049
**Yes**	Ref	Ref	Ref

~ Mild disease category was excluded because it had no observed cases.

**Table 3:** Logistic regression model comparing mortality between the first and second wave and other predictors.

	Unadjusted Model			Adjusted Model		
Effect	OR	95% C I		OR	95% C I	
**Wave**						
2nd wave	0.63	0.414	0.960	1.733	0.645	4.655
1^st^ wave	Ref			Ref		
**Age**				1.048	1.021	1.074
**Number of Comorbidities**				0.997	0.806	1.234
**Length of Stay**				0.961	0.936	0.986
**Therapeutic Anticoagulation**						
Yes				0.871	0.372	2.038
No				Ref		
**Steroids**						
Yes				2.316	0.859	6.246
No				Ref		
**Convalescent Plasma**						
Yes				1.350	0.605	3.012
No				Ref		
**Mechanical Ventilation**						
Yes				0.048	0.020	0.114
No				Ref		

**c statistic for unadjusted model**– 0.947.

**c statistic for adjusted model**– 0.947.

## Discussion

In our study comparing the outcomes of patients between the first and second wave, we found that the odds of dying was higher in the first wave compared to the second wave. However, in the fully adjusted model the odds of death were not significantly different between the two waves.

The use of steroids was significantly higher in the second wave of COVID-19. This can be attributed to the body of research which accumulated over time as to the effectiveness of steroid use in treatment of COVID-19 [22,23]. In the past 50 years, the potential benefit of corticosteroids in treating sepsis or acute respiratory distress syndrome (ARDS) has been evaluated in many randomized controlled trials [24,25]. Much of the initial guidance issued by medical societies concerning steroid use in COVID-19 was extrapolated from studies that used steroids to treat severe acute respiratory syndrome (due to SARS-Co-V) [18].

In the early days of COVID-19, a cohort study conducted in Wuhan China found that among a subgroup of 84 patients with COVID-19 and ARDS, treatment with methylprednisolone was associated with a lower risk of death (hazard ratio 0.39, 95% CI, 0.20–0.72) [26]. The results of the RECOVERY trial was one of the more robust studies of steroid use in COVID-19 were published in July 2020 [27], the findings showed that dexamethasone 6 mg once daily (intravenous or by mouth) for up to 10 days reduced 28-day mortality (rate ratio [RR] 0.83, 95% CI 0.75–0.93) in hospitalized patients with COVID-19. Also, among those receiving oxygen, the risk of progression to invasive mechanical ventilation or death was lower in the dexamethasone group [27]. This study also recommended hypoxemic COVID-19 patients should receive dexamethasone given the reduced risk of death and increased likelihood of hospital discharge, based on evidence at the time. Following this study in July 2020, steroids became a mainstay of treatment for hospitalized patients for COVID-19 as is also evidenced in our study by the high number of patients who received steroids in the second wave (October 2020 -January 2021) compared to the first wave (April 2020 -June 2020).

Intubation rates were noticed to be higher in the first wave in this study. Earlier on, some experts advocated for the avoidance of non-invasive ventilation (i.e., proceeding to early intubation if escalating beyond 6 L/min with continued hypoxemia or increased work of breathing). This was predicated on an increased risk of aerosolization and high likelihood that patients who need these modalities will ultimately, rapidly deteriorate and require mechanical ventilation (e.g., within one to three days) [28,29]. However later in the pandemic the use of non-invasive ventilation (NIV) was more prevalent, which may have contributed to lower intubation rates [30]. Older age has been shown to be a predictor of intubation in COVID-19 in some studies and since the population affected in the second wave was younger, this may be why intubation rates were higher in the first wave in this study [31,32].

The average length of hospital stay was noted to be longer in the first wave. The greater availability of pharmacological interventions was likely a major contributory factor to earlier discharge home in the second COVID-19 wave–such as Remdesivir, monoclonal antibodies and steroids. In some studies, Remdesivir was shown to save hospitals $US12,000 per patient by shortening hospital length of stay (LOS) by 4 days. This presumed benefit was extrapolated from the ACTT-1 trial [33], which found median time to recovery (an approximation of median time to hospital discharge) of 11 days in patients receiving Remdesivir versus 15 days in those receiving placebo [34].

Another factor may be the different criteria for admission and discharges between the first and second waves, which changed as we gained further knowledge regarding the natural history of COVID-19 infection [35,36].

Some studies have shown risk factors which account for longer length of hospital stay to be, older age and greater number of comorbidities (both of which were higher in the first wave) [37].

A multi-mechanism approach involving the use of steroids, anticoagulants, viral suppression, and nonpharmacological interventions such as home oxygen in COVID-19 patients was shown in one study to reduce length of hospital stay. This study showed that these interventions decreased the average ICU length of stay by 5.4 days [38].

The provision of home oxygen to patients with COVID-19, which became much more widely available during the second wave of the pandemic, also greatly facilitated earlier discharge from hospitals [39].

## Limitations

Interpretation of our findings might be limited by the sample size of this study. It is also limited as it is a single center study so the results may not be generalizable to other institutions.

Laboratory results were not analyzed, which could contribute as additional comparative factors between the 2 waves and explain better the reason for some of the differences in the 2 waves.

Analysis of the different COVID-19 strains was not completed as part of this study. This could be an important factor explaining differences in demographics and outcomes between the 2 waves. Data on behavioral risk factors such as smoking, alcohol and illicit drug use was not gathered, these can potentially impact outcomes. Another limitation would be the difference in timing of the surges, wave one occurring in the spring and wave two in the fall to winter seasons a time associated with increased incidence of viral upper respiratory infections.

## Conclusion

The result of our study demonstrates that the odds of mortality were less in the second wave compared to the first wave, however, in the fully adjusted model there was no significant difference between the two waves. Our findings also suggest that there was increased steroid use, less mechanical intubations, early discharge home with oxygen in the second wave compared to the first wave, which potentially improved outcomes. Future studies may consider examining the individual influence of each of these measures on the cumulative beneficial effect.
